# A sampling, exposure and receptor framework for identifying factors that modulate behavioural responses to disturbance in cetaceans

**DOI:** 10.1111/1365-2656.13787

**Published:** 2022-08-09

**Authors:** Cormac G. Booth, Naomi Brannan, Rebecca Dunlop, Ari Friedlander, Saana Isojunno, Patrick Miller, Nicola Quick, Brandon Southall, Enrico Pirotta

**Affiliations:** ^1^ SMRU Consulting, Scottish Oceans Institute University of St Andrews St Andrews UK; ^2^ Southeast Asia Marine Mammal Research Hong Kong Hong Kong; ^3^ Cetacean Ecology and Acoustics Laboratory Moreton Bay Research Station and School of Biological Sciences University of Queensland Brisbane Australia; ^4^ Southall Environmental Associates, Inc. Aptos California USA; ^5^ University of California, Institute of Marine Science Santa Cruz California USA; ^6^ Sea Mammal Research Unit, Scottish Oceans Institute University of St Andrews St Andrews UK; ^7^ School of Biological and Marine Sciences University of Plymouth Plymouth UK; ^8^ Nicholas School of the Environment Duke University Beaufort North Carolina USA; ^9^ Centre for Research into Ecological and Environmental Modelling University of St Andrews St Andrews UK

**Keywords:** anthropogenic disturbance, behavioural responses, marine mammals, modulating factors, underwater noise

## Abstract

1. The assessment of behavioural disturbance in cetacean species (e.g. resulting from exposure to anthropogenic sources such as military sonar, seismic surveys, or pile driving) is important for effective conservation and management. Disturbance effects can be informed by Behavioural Response Studies (BRSs), involving either controlled exposure experiments (CEEs) where noise exposure conditions are presented deliberately to meet experimental objectives or in opportunistic contexts where ongoing activities are monitored in a strategic manner. In either context, animal‐borne sensors or in situ observations can provide information on individual exposure and disturbance responses.
2. The past 15 years of research have greatly expanded our understanding of behavioural responses to noise, including hundreds of experiments in nearly a dozen cetacean species. Many papers note limited sample sizes, required knowledge of baseline behaviour prior to exposure and the importance of contextual factors modulating behavioural responses, all of which in combination can lead to sampling biases, even for well‐designed research programs.
3. It is critical to understand these biases to robustly identify responses. This ensures outcomes of BRSs help inform predictions of how anthropogenic disturbance impacts individuals and populations. Our approach leverages concepts from the animal behaviour literature focused on helping to avoid sampling bias by considering what shapes an animal's response. These factors include social, experience, genetic and natural changes in responsiveness.
4. We developed and applied a modified version of this framework to synthesise current knowledge on cetacean response in the context of effects observed across marine and terrestrial taxa. This new ‘Sampling, Exposure, Receptor’ framework (SERF) identifies 43 modulating factors, highlights potential biases, and assesses how these vary across selected focal species.
5. In contrast to studies that identified variation in ‘Exposure’ factors as a key concern, our analysis indicated that factors relating to ‘Sampling’ (e.g. deploying tags on less evasive individuals, which biases selection of subjects), and ‘Receptor’ (e.g. health status or coping style) have the greatest potential for weakening the desired broad representativeness of BRSs. Our assessment also highlights how potential biases could be addressed with existing datasets or future developments.

## Graphical abstract

This paper helps disentangle potential biases and modulating factors to help improve behavioural response studies for whales and dolphins.

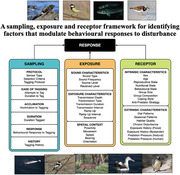

## 1 UNDERSTANDING RESPONSES TO DISTURBANCE

Marine ecosystems are subject to a variety of natural and anthropogenic stressors, such as human‐induced climate change, variable prey resources, altered habitat and oceanographic regimes, fisheries interactions (e.g. bycatch), marine contaminants and biotoxins. All of these potential stressors are capable of impairing the health and fitness of marine species through single or combined processes (National Academies of Sciences Engineering and Medicine, 2017; Pirotta, Booth, et al., 2022; Pirotta, Thomas, et al., 2022). In recent decades, there has been considerable interest in assessing the effects of anthropogenic noise on marine ecosystems (e.g. from military sonar, seismic surveys, pile driving, vessel traffic) as sound plays a critical role in the lives of many marine species (e.g. Duarte et al., 2021; Hawkins & Popper, 2017; Kunc & Schmidt, 2019; Stanley et al., 2012; Tablado & Jenni, 2017). Some marine mammals are considered particularly sensitive to chronic and acute exposure to noise because of their strong reliance on sound production and reception for their life functions (Erbe et al., 2019).

Exposure to human activities can cause changes in the behaviour and physiology of individual animals across a wide range of taxa (Ames et al., 2020; Beale & Monaghan, 2004b; Frid & Dill, 2002). Such changes can affect an individual's vital rates, such as survival and reproduction, via several pathways (e.g. energy stores, immune status and stress hormones) described in the Population Consequences of Disturbance framework (Pirotta et al., 2018, 2021). Effective management of protected species requires understanding responses to human disturbance(s) in the context of their effects on individual vital rates (e.g. survival or reproduction) and population dynamics (Ames et al., 2020; Sutherland, 1996). Quantifying population‐level consequences of disturbance and the influence of any modulating factors is critical to correctly identify the species in the greatest need of conservation (Beale & Monaghan, 2004a; Gill et al., 2001).

‘Stimulus–response’ methods (i.e. those where a receptor is presented with a stimulus and a potential response is monitored) have long been a feature of behavioural research for almost a century (Lorenz, 1937; Shettleworth, 2001), and, with more recent technological development, they have become an important tool for quantifying behavioural responses of wildlife to human‐induced disturbance (Harris et al., 2018; Shannon et al., 2016; Southall et al., 2016, 2021). In marine mammals, responses to anthropogenic disturbance have been quantified using Behavioural Response Studies (BRSs), involving either controlled exposure experiments (CEEs) where noise exposure conditions are presented deliberately to meet experimental objectives (i.e. animals are exposed to a controlled level or ‘dose’ of a potential stressor) or in opportunistic contexts where ongoing activities are monitored in a strategic manner (e.g. DeRuiter et al., 2013; Falcone et al., 2017; Tyack et al., 2011; Williams et al., 2002). In either context, behaviour can be monitored using animal‐borne sensors (Dunlop et al., 2013; Isojunno et al., 2016; Johnson & Tyack, 2003; Southall et al., 2012, 2017) and/or remote observations (e.g. Bejder et al., 2006; Durban et al., 2022; Scheidat et al., 2004). These methods have been extensively deployed in observational and controlled studies over the past 15 years, including hundreds of experiments in nearly a dozen species (reviewed by Harris et al., 2018; Nowacek et al., 2016; Southall et al., 2016). BRSs (including CEEs) on free‐living marine mammals have significant logistical and analytical challenges (Southall et al., 2016). For example, many papers acknowledge limited sample sizes and short baseline pre‐exposure periods (Falcone et al., 2017; Friedlaender et al., 2016; Jensen et al., 2011).

The collective body of research has highlighted that while received noise is certainly an important aspect of response type and probability in some scenarios, the behavioural responses of marine mammals to sound disturbance cannot be determined solely from acoustic metrics (Gomez et al., 2016; Southall et al., 2007). Additionally, sampling biases and contextual variables have an ‘equal or greater importance for determining the form, probability and severity of a response’ (Ellison et al., 2011). Just as a mixture of breadth and depth of knowledge is necessary for human innovation (Boh et al., 2014), interdisciplinary collaboration is fundamental to understanding ecological systems at the scales at which they are managed (Goring et al., 2014). The use of frameworks to provide structure in framing and understanding a problem is not new to science (e.g. Diefes‐Dux et al., 2004; Tonn, 1990; Wilson et al., 2020). In the domain of animal behaviour, Webster and Rutz (2020) detailed a cross‐taxon framework for avoiding sampling biases in both laboratory and field studies, which has informed guidelines to improve reporting standards in animal behavioural research (Rutz & Webster, 2021). Consequently, to understand marine mammal behavioural responses we believe it is beneficial to look beyond taxon‐specific research to identify biases or modulating factors accounted for in more easily studied taxa. Here we have taken a wider perspective, across taxa, to help understand what is achievable and has the potential to help translate the observed responses from BRSs to effective conservation and management.

## 2 THE ‘SAMPLING, EXPOSURE, RECEPTOR FRAMEWORK’

**FIGURE 1:**
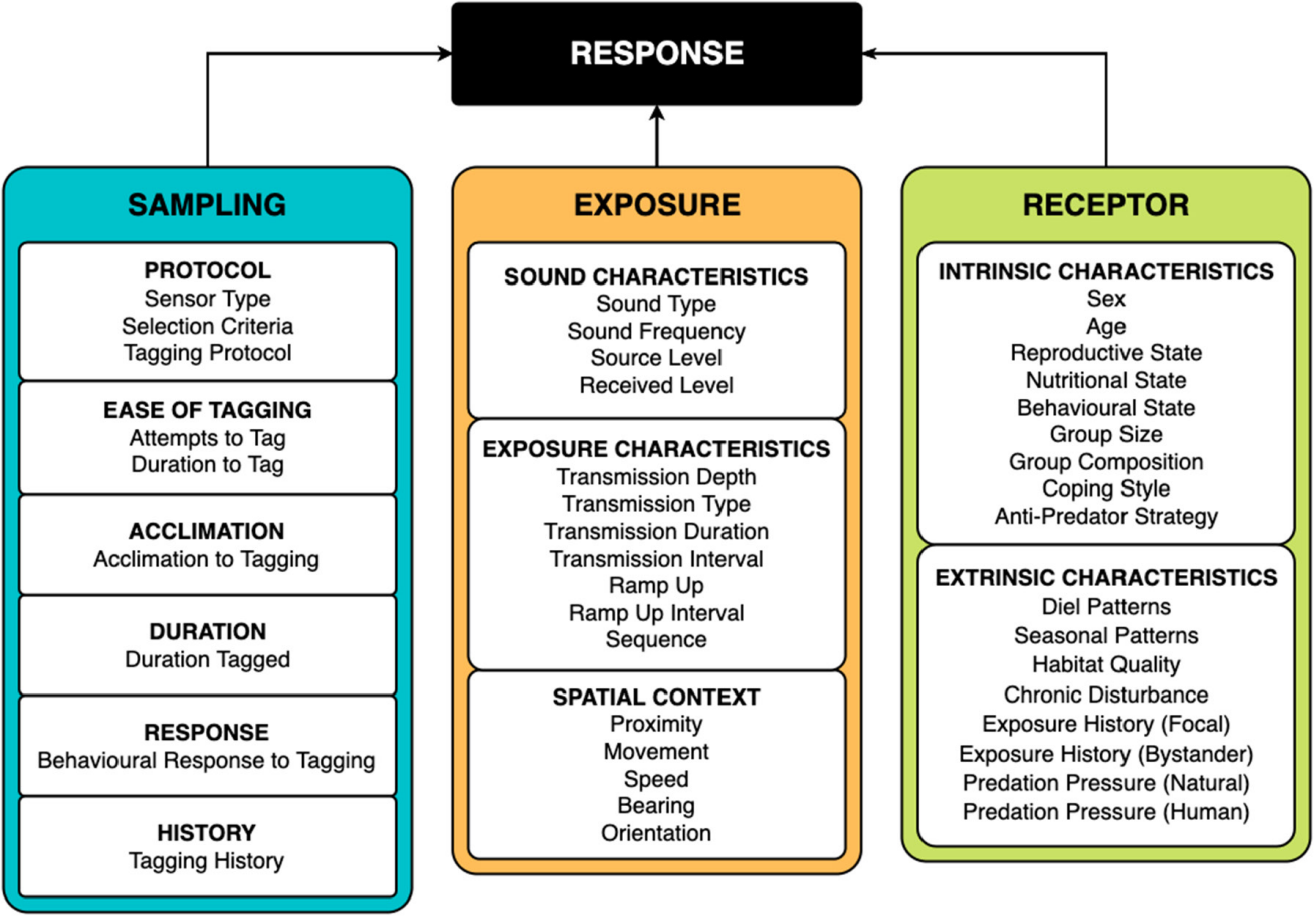
Schematic outlining the factors contributing to a behavioural response. Note that it is likely interactions occur across factors from different categories.

A literature review of marine mammal BRSs (principally, but not limited to CEEs) was performed to identify factors that could modulate behavioural responses to disturbance. A subsequent, wider literature review was then performed to identify modulating factors that are well‐established in other (non‐marine mammal) taxa (Figure 1). We summarised these reviews into a framework (see Supplementary Information SI S1) to identify 43 candidate modulating factors within a CEE, which are grouped into three categories, encompassing the Sampling, Exposure and Receptor Framework (SERF) (Table 1). In this framework, ‘Sampling’ refers to the selection of study animals and how data are collected from them. ‘Exposure’ refers to the parameters of the potentially disturbing stimulus to which focal animals are exposed. ‘Receptor’ refers to intrinsic and extrinsic characteristics of the study animals themselves. Each factor of the framework was then assessed by a panel of marine mammal experts (the paper's co‐authors) to indicate whether it has been, or could be, addressed with existing CEEs data and to highlight where future research may be insightful (a template SERF is available in SI S2). We focused the assessment on CEEs to allow appropriate consideration of ‘Exposure’ which is controlled for in these experiments and we comment on the application of this framework to more opportunistic BRSs in the Discussion.

**TABLE 1:** Sampling‐Exposure‐Receptor framework (SERF). Further details for each category and factor are provided in SI S1, and a template is available in SI S2

Category	Subcategory	Factor	Example
Sampling	Protocol	Sensor type	Mul et al. (2019)
		Selection criteria	Barrett‐Lennard et al. (1996)
		Tagging protocol	Garamszegi et al. (2009)
	Ease of tagging	Attempts taken to tag	Schuert et al. (2021)
		Time taken to tag	
	Acclimation	Acclimation to tagging	Weimerskirch et al. (2002)
	Duration	Duration tagged	Morellet et al. (2009)
	Response	Behavioural response to tagging	Sun et al. (2015)
	History	Tagging history	Van Oers and Carere (2007)
Exposure	Sound characteristics	Sound type	Bejder et al. (2009), Ellison et al. (2011, 2018), Harris et al. (2018) and Southall et al. (2007, 2012, 2016, 2019)
		Sound frequency	
		Source level	
		Received level	
	Exposure characteristics	Duration	
		Transmission depth	
		Transmission type	
		Transmission duration	
		Transmission interval	
		Ramp up	
		Ramp up interval	
		Sequence	
	Spatial context of exposure	Proximity	DeRuiter et al. (2013)
		Movement	Fiori et al. (2019)
		Speed	Ng and Leung (2003)
		Bearing	Buck and Tyack (2000) and Clark et al. (1999)
		Orientation	
Receptor	Intrinsic	Sex	Williams et al. (2002)
		Age	Berger et al. (2007)
		Reproductive state	Andersen and Aars (2007)
		Nutritional state	Beale and Monaghan (2004a)
		Behavioural state	Goldbogen et al. (2013)
		Social context—group size	Jarrett et al. (2020)
		Social context—group composition	Stamation et al. (2010)
		Coping style	Naguib et al. (2013)
		Anti‐predator strategy	Isojunno et al. (2016)
	Extrinsic	Diel patterns	Caruso et al. (2020)
		Seasonal patterns	Andersen et al. (2012)
		Habitat quality	Beale and Monaghan (2004a)
		Chronic disturbance	Walker et al. (2005)
		Exposure history—focal animal	Sun et al. (2015)
		Exposure history—bystander	
		Predation pressure—natural	St Clair et al. (2010)
		Predation pressure—human	Stankowich (2008)

## 3 SAMPLING

The ‘Sampling’ category encompasses biases that may arise from the selection of study animals and how data are collected from them, arranged into six subcategories: Protocol, Ease of tagging, Acclimation, Duration, Response and History. We term amenability to tag deployment as ‘Ease of tagging’, influenced by species and individual behavioural characteristics such as responsiveness to vessel approach and surface behaviour. CEEs typically involve tagging individual animals with sensors and selecting which sensors to use and which individuals to tag can lead to inherent biases. Some of these biases may be unintentional (e.g. targeting individuals that are most tolerant of being approached), deliberate (e.g. targeting larger individuals to increase tagging success) or unavoidable (e.g. targeting robust adults rather than juveniles or less healthy individuals for ethical or permitting reasons). For example, older female northern killer whales, *Orcinus orca*, have been described as more difficult to approach closely for biopsy than males (Barrett‐Lennard et al., 1996). Sensor type (e.g. DTAGs, satellite tags) determines how and where sensors are attached, which can influence movement and behaviour inferences at a fine scale (Mul et al., 2019).

Tagging protocols may sample non‐randomly from populations—for example, in collared flycatchers, *Ficedula albicollis*, ‘trappability’ is correlated with boldness (Garamszegi et al., 2009). Ideally, BRS protocols should minimise potential ‘trappability’ biases, although this is challenging for wild marine mammal populations. Assessing an individuals' ‘ease of tagging’, that is, the number of attempts or time taken to tag, could facilitate investigation of how baseline/response metrics differ (Schuert et al., 2021). Similarly, it is important to quantify responses to tagging which, in marine mammals and other taxa, have modulated subsequent responses to stressors (Miller et al., 2009; Sun et al., 2015). Individuals may recover from tagging, capture and handling at different rates (ranging from minutes to days). For example, male wandering albatrosses, *Diomedea exulans*, react more strongly to handling than females, but recover more quickly (Weimerskirch et al., 2002). In roe deer, *Capreolus capreolus*, individuals fitted with a GPS collar showed significant changes in baseline behaviour for several days after capture and handling, preferring refuge habitat, avoiding sources of human disturbance, and showing reduced activity levels (Morellet et al., 2009). Therefore, definition and justification of acclimation periods should be clear and appropriate (e.g. using fixed‐time or behaviour‐based criteria). Finally, tagging history is important, as repeated tagging, capture and handling experiences, either successful or attempted, can alter responses over time by increasing or decreasing sensitivity to stressors (Van Oers & Carere, 2007).

## 4 EXPOSURE

The ‘Exposure’ category consists of 17 factors arranged into three subcategories: Sound Characteristics, Exposure Characteristics and Spatial Context of Exposure. CEEs involve exposing individual animals to sound stimuli; the characteristics of the stimulus, how individuals are exposed, and the spatial context of exposure all have the potential to modulate behavioural responses. Stimulus‐related modulating factors that have been well established include sound type (e.g. Low Frequency Active Sonar, Mid Frequency Active Sonar), frequency, source level and received level (Ellison et al., 2011, 2018; Harris et al., 2018; Southall et al., 2007, 2012, 2016, 2019). Factors that concern the experimental design of how each subject is exposed include the total duration of the controlled exposure, transmission depth, transmission type (e.g. continuous or intermittent), the duration of and interval between each transmission, whether the stimulus level was gradually increased (ramped up), how it was ramped up and, if multiple stimuli were used, the sequence in which those stimuli were delivered (Isojunno et al., 2020; Kok et al., 2018). Factors pertaining to the spatial context of exposure include the proximity of the individual to the stimulus (DeRuiter et al., 2013), whether the stimulus was moving towards or away from the individual (Fiori et al., 2019), the speed of the stimulus approach (Ng & Leung, 2003), the bearing of the stimulus and the orientation of the individual (Buck & Tyack, 2000; Clark et al., 1999).

## 5 RECEPTOR

Modulating factors associated with the receptor, the study subject animal, can be broken down into 17 intrinsic and extrinsic factors. Intrinsic factors such as sex, age, behavioural state, reproductive state and nutritional state all have the potential to modulate responses to disturbance. For example, male and female northern resident killer whales use different avoidance tactics when approached by vessels. Both respond by moving less predictably, but females tend to reduce surfacing predictability, whereas males tend to reduce directional predictability (Williams et al., 2002). Responses can also be age dependent. For example, juvenile marine iguanas, *Amblyrhynchus cristatus*, are more sensitive to disturbance than adults, with age acting as a strong predictor of flight initiation distance (Berger et al., 2007). Individuals in different reproductive and nutritional states can also be more sensitive and react more strongly to disturbance (Andersen & Aars, 2007; Beale & Monaghan, 2004a). Similarly, some cetaceans in different behavioural states and/or environmental contexts may respond differently to the same stimulus (Friedlaender et al., 2016; Goldbogen et al., 2013; Southall et al., 2019).

Extrinsic factors, such as whether individuals are tested alone or with conspecifics, especially for social species, can modulate the severity of responses (Durban et al., 2022; Visser et al., 2016; Wascher et al., 2008). Individuals in larger groups, or groups with calves, can also be more sensitive to disturbance, as observed in humpback whales, *Megaptera novaeangliae*, and waterbirds (Jarrett et al., 2020; Stamation et al., 2010). Other important considerations include coping style and anti‐predator strategies. For example, responses to acoustic disturbance in nesting great tits, *Parus major*, appear to be more dependent on personality and sex than noise characteristics (Naguib et al., 2013), where ‘bolder’ parents were less affected by noise disturbance, returning to their nest boxes sooner than ‘shyer’ parents. Anti‐predator strategies or predation risk itself (St Clair et al., 2010; Stankowich, 2008) has been demonstrated to influence behavioural responses to disturbance (Frid & Dill, 2002; Isojunno et al., 2020; Miller et al., 2022).

In non‐marine mammal taxa, temporal factors such as diel (e.g. whether a species is nocturnal or diurnal) and seasonal (e.g. whether an individual is breeding or moulting) patterns can modulate sensitivity to disturbance, the severity of response, and the likelihood of exposure (Caruso et al., 2020). Some fish species have been observed to respond more strongly to disturbance at night (Neo et al., 2018). In marine mammals, distinct diel habits are poorly understood, but distinct temporal patterns in foraging have been observed (e.g. Linnenschmidt et al., 2013). BRSs are typically only carried out in daylight hours and are limited by weather conditions and restrictions set by permit requirements, all of which can also affect sampling. Individuals during the breeding season may be more sensitive and react more strongly to disturbance than during the non‐breeding season. For example, breeding harbour seals, *Phoca vitulina*, are less alert to, less likely to flee, and quicker to return to a haul‐out site when approached by humans or vessels than during pre‐ and post‐breeding seasons (Andersen et al., 2012). Spatial factors, such as habitat quality, can also influence sensitivity to disturbance and the severity of responses. Individuals in higher‐quality habitats may be able to afford interruptions to critical behaviours (e.g. foraging), particularly if they are in a condition to respond to disturbance. This has been observed in Ruddy turnstones, *Arenaria interpres*, (Beale & Monaghan, 2004a) where animals in enhanced condition (via the experimental setup) exhibited greater responsiveness to disturbance and flew away to greater distances. In addition, these animals demonstrated more frequent scanning for predators. However, in northern bottlenose whales, *Hyperoodon ampullatus*, no such pattern was observed, and low energy stores were not correlated with simulated predator avoidance (Siegal et al., 2022). Conversely, chronic disturbance or past exposure to experimental conditions, either as a focal animal or as a bystander, can result in habituation or desensitisation to stressors. For example, desensitisation has been observed via lower behavioural response scores to a subsequent stressor in European badgers, *Meles meles*, (Sun et al., 2015). Magellanic penguin chicks, *Spheniscus magellanicus*, raised in tourist‐visited areas of a breeding colony are also less likely to flee when approached by humans than chicks raised in areas not visited by tourists (Walker et al., 2005).

## 6 APPLYING THE FRAMEWORK

The framework was applied to seven cetacean species for which CEEs have been conducted: blue whales, *Balaenoptera musculus*; humpback whales; sperm whales, *Physeter macrocephalus*; northern bottlenose whales; long‐finned pilot whales, *Globicephala melas*; short‐finned pilot whales, *Globicephala macrorhynchus*; and Cuvier's beaked whales, *Ziphius cavirostris* (refer to SI S1 for details). For every factor of the framework, each species was allocated a value between 1 to 3 indicating whether modulating factors have been, or could be, assessed and accounted for in a CEE. These assessment values ranged from ‘Yes, from published or unpublished data’ (1) to ‘Possibly, from the re‐analysis of existing data’ (2) to ‘No, because data have not (yet) been collected or it's not (currently) feasible to collect such data’ (3). Assessments were conducted by a group of marine mammal scientists with expertise in CEEs (Table 2). It should be noted that the SERF assessment values are not intended to provide a review of the status of marine mammal BRSs or CEEs, which is beyond the scope of this Concept.

**TABLE 2:** Sampling‐Exposure‐Receptor framework (SERF) assessment values for focal species of marine mammal controlled exposure experiment studies. Key: 1 = yes, from published or unpublished data. 2 = possibly, from the re‐analysis of existing data. 3 = no because the data have not (yet) been collected or it is not currently feasible to collect such data. Refer to S1 for details on study species and populations

Category	Subcategory	Factor	Blue whale (*Balaenoptera musculus*)	Humpback whale (*Megaptera movaeangliae*)	Sperm whale (*Physeter macrocephalus*)	Cuvier's beaked whale (*Ziphius cavirostris*)	Northern bottlenose whale (*Hyperoodon ampullatus*)	Long‐finned pilot whale (*Globicephala melas*)	Short‐finned pilot whale (*Globicephala macrorhynchus*)
Sampling	Protocol	Sensor type	1	1	1	1	1	1	1
		Selection criteria	1	1	1	1	1	1	1
		Tagging protocol	1	1	1	1	1	1	1
	Ease of tagging	Attempts taken to tag	2	1	2	2	3	2	2
		Time taken to tag	2	1	1	2	3	2	2
	Acclimation	Acclimation to tagging	1	1	1	1	1	1	1
	Duration	Duration tagged	1	1	1	1	1	1	1
	Response	Behavioural response to tagging	2	1	2	1	2	2	1
	History	Tagging history	2	1	2	1	3	2	2
Exposure	Sound Characteristics	Sound type	1	1	1	1	1	1	1
		Sound frequency	1	2	1	1	1	1	1
		Source level	1	2	1	1	1	1	1
		Received level	1	1	1	1	1	1	1
	Exposure Characteristic	Duration	1	1	1	1	1	1	1
		Transmission depth	1	1	1	1	1	1	1
		Transmission type	1	1	1	1	1	1	1
		Transmission duration	1	1	1	1	1	1	1
		Transmission interval	1	1	1	1	1	1	1
		Ramp‐up	1	1	1	1	1	1	1
		Ramp‐up interval	1	1	1	1	1	1	1
		Sequence	1	1	1	1	1	1	1
	Spatial Content of Exposure	Proximity	1	1	1	1	1	1	1
		Movement	1	1	1	1	1	1	1
		Speed	1	1	1	1	1	1	1
		Bearing	1	1	1	1	1	1	1
		Orientation	1	1	2	2	2	2	2
Receptor	Intrinsic	Sex	3	1	1	2	2	3	2
		Age	1	1	2	2	2	1	2
		Reproductive state	3	1	1	3	2	3	3
		Nutritional slate	3	1	1	3	1	1	3
		Behavioural stale	1	1	1	1	2	1	1
		Social context—group size	1	1	1	1	1	1	1
		Social content—group composition	1	1	1	2	1	1	2
		Coping style	3	3	3	3	3	3	3
Anti‐predator strategy	2	1	1	1	1	1	1
Extrinsic	Diel patterns	3	3	1	1	2	1	1
	Seasonal patterns	1	1	3	1	2	3	2
	Habitat quality	1	3	3	1	3	3	1
	Chronic disturbance	2	1	3	1	1	3	1
	Exposure history—focal animal	2	1	2	1	3	2	1
	Exposure history—bystander	2	1	3	2	3	3	2
	Predation pressure—natural	2	2	2	2	2	2	2
	Predation pressure—human	2	1	2	1	2	2	2

## 7 DISCUSSION

The SERF assessment indicated that the existing marine mammal literature has or could in the future effectively account for Sampling and Exposure factors (i.e. given the approaches currently used in BRSs). Experiments on humpback whales indicated the fewest data gaps, whilst CEEs on northern bottlenose whales indicated the most. Many of these differences highlighted by the SERF assessment are likely a consequence of differing methods for studying those target species. For example, northern bottlenose whales had many gaps identified because tagged animals were those individuals that were most likely to approach the research vessel (Siegal et al., 2022), but a lack of identifying features made it impossible to evaluate whether individuals approaching the research vessel were representative of the population. Photo‐identification catalogues are improving, which might make integration of such datasets possible in the future, to improve the scope for inference. For some species, smaller populations, or populations with high site fidelity (in the study area) may also make it easier to follow focal individuals and determine their age and sex class. Ultimately, greater long‐term baseline data on study populations is required to help understand patterns observed in BRSs.

Sampling factors are partially or fully within the control of researchers (e.g. sensor type), can be addressed by designing them into the scope of a CEE (e.g. acclimation to tagging) or do not require additional methodology (e.g. observing behavioural responses to tagging). Nearly all the Exposure factors can be addressed with published or unpublished data, as these modulating factors have historically been the primary focus of CEEs.

The Receptor category included the most factors that have not been addressed, as these factors often cannot be resolved through a CEE (e.g. diel or seasonal patterns) and require additional information or long‐term baseline datasets to inform about the study population (e.g. biopsies to determine sex or reproductive state) or are difficult to quantify in free‐living populations (e.g. coping style). For species that range over large areas or migrate, factors such as habitat quality, chronic disturbance or the effects of multiple stressors (Pirotta, Booth, et al., 2022; Pirotta, Thomas, et al., 2022) and predation pressure (Wirsing et al., 2008) are challenging to fully address, as they can influence study animals far beyond the window of observation (i.e. beyond the CEE).

Although most CEEs focus on the behavioural response of the study animal, physiological responses should also be considered. Studies on American black bears, *Ursus americanus*, highlighted significant increases in heart rate when approached by unmanned aerial vehicles (UAVs) but little to no behavioural response (Ditmer et al., 2015). Similar discrepancies between behavioural and physiological responses have been observed in harbour porpoise in managed care (Elmegaard et al., 2021), suggesting that the absence of a behavioural response does not indicate the absence of any response (Bejder et al., 2009). Currently, such responses are being monitored more in wild, free‐living marine mammals as advancements in remote sampling and non‐invasive telemetry show promise for future monitoring (e.g. Aoki et al., 2021; Elmegaard et al., 2021; Goldbogen et al., 2019; McKnight et al., 2019; Siegal et al., 2022).

The modulating factors in SERF, particularly in the Sampling and Receptor categories, are dependent on the ability to identify individuals, which may be challenging during experiments for numerous reasons. For example, it is not possible to quantify ease of tagging (either as attempts or time taken to tag) or tagging history in sperm whales, northern bottlenose whales and long‐finned pilot whales, because individuals are not typically identifiable prior to tagging. Orientation with respect to the source at the moment of exposure has been explored using high‐resolution telemetry (Isojunno et al., 2021) but may be important to better understand at the group level for more social species which form large aggregations or coordinated foraging at depth (e.g. short‐finned pilot whales; Visser et al., 2016). Many factors interact within and across categories of SERF. For example, sensor type will dictate tagging protocol and how individuals are approached. Acclimation to tagging and duration of tagging period are dependent on the attachment life or durability of a sensor, which can differ between tag types, ranging from hours (e.g. DTAGs) to days (e.g. satellite tags). Sex and/or age often influence selection criteria for tagging. Adult male Cuvier's beaked whales can be distinguished in the field due to lighter body coloration and the presence of teeth, but adult females and subadult males are difficult to tell apart. This can lead to animals of presumed known and unknown sex being tagged. However, adult males can often be easier to tag due to their frequent location on the periphery of groups making them more available to tagging (and calves or females with calves are not typically tagged (Quick, pers. comm.)).

## 8 CONCLUSIONS AND NEXT STEPS

Understanding the influence of contextual variables or modulating factors on the probability of response is crucial for the interpretation of BRS results (Pirotta et al., 2021; Southall et al., 2016, 2019). This study explores these factors by outlining a framework that aids the critical assessment of variables that can modulate behaviour responses during BRSs. While the SERF assessment has focused on marine mammal CEEs, many existing studies (particularly opportunistic BRSs where large sample sizes have been amassed and often better baseline information on the population is available, e.g. Falcone et al., 2009, 2017) have employed a variety of non‐CEE approaches to explore potential disturbance from military sonar and other noise stressors, such as the effects of vessel noise, seismic surveys and offshore wind farm construction (e.g. Dunlop et al., 2016; Graham et al., 2019; Mikkelsen et al., 2019; Pirotta et al., 2012). Such studies could also benefit from considering some of the modulating factors highlighted using the SERF assessment (Table 3).

**TABLE 3:** Key issues highlighted by the SERF assessment and suggested next steps to advance the understanding of behavioural responses in marine mammals

Key issue	Suggested steps
Many ‘Sampling’ modulating factors are challenging to address without supporting Information	Collection of long‐term baseline information to understand the study population and to help identify modulating factors
Current SERF assessment is limited to specific CEEs (in which ‘Exposure’ is controlled)	Carry out SERF assessment on wider CEE and BRS research (including opportunistic studies where large sample sizes and baseline knowledge may help elucidate ‘Sampling’ and ‘Receptor’ factors)
Many factors are poorly understood using current BRS designs	Revisit past BRS datasets to apply SERF and quantify population‐specific knowledge gaps Design future BRS to address specific modulating factors
Modulating factors may help in translating from responses to population‐ level effects	Greater interaction between BRS and PCoD analysts to determine what factors could be most crucial in extrapolating BRS results outside the experimental setting—to determine which factors are most crucial to outcomes

Building on the assessments made here, a natural next step would be to consider existing CEE datasets (and data from other marine mammal BRSs) to establish where modulating factors may have not been controlled for (e.g. individual ease of tagging). Significant logistical challenges remain in studying free‐ranging marine mammal species and quantifying their responses to stressors. Future CEEs could be designed to explicitly explore some of the more accessible modulating factors and key sensitivities described here, particularly to help understand responses in the context of population level effects (e.g. Pirotta, Booth, et al., 2022; Pirotta, Thomas, et al., 2022).

It is important to note that Webster and Rutz (2020) highlight that ‘the modulating factors are not problematic by themselves. They are often the focus of well‐designed research projects or are confounding factors that have been explicitly controlled for. Concerns arise whenever samples of study subjects are unwittingly biased with regards to any of these categories, and when researchers overlook that fact’. As such, modulating factors are an embedded part of any exhibited behaviour and understanding the role of such factors can help improve assessments of anthropogenic impacts on individuals and populations. The consideration and integration of modulating factors into future CEEs could lead to a step change in our understanding of responses to noise and other anthropogenic stressors against the backdrop of a changing environment.

## AUTHOR CONTRIBUTIONS

Cormac G. Booth and Naomi Brannan conceived the ideas; Cormac G. Booth led the manuscript and Naomi Brannan and Cormac G. Booth developed the initial framework and Tables; Patrick Miller, Saana Isojunno, Brandon Southall, Ari Friedlander, Nicola Quick, Rebecca Dunlop applied the SERF to existing BRS datasets. All authors contributed critically to the manuscript.

## CONFLICT OF INTEREST

The authors declare no conflict of interest.

## Supporting information


Data S1

Data S2

## Data Availability

No data were used for this manuscript.
