# Supplementary material for: A sampling, exposure and receptor framework for identifying factors that modulate behavioural responses to disturbance in cetaceans
Source: J Anim Ecol. 2022 Aug 9;91(10):1948–60. doi: 10.1111/1365-2656.13787 (PMC9804311; doi:10.1111/1365-2656.13787)
Supplement: Supplementary file 1 — Data S1 [file JANE-91-1948-s001.docx]

**Supplementary Information 1**

We present a framework to identify potentially modulating factors within a CEE consisting of 43 factors that are grouped into three categories encompassing the “Sampling, Exposure and Receptor” Framework (“SERF”) and each is described below.

**Sampling**

**Sensor Type:** Tag positioning on an adult male killer whale, *Orcinus orca*, influenced movement and behaviour inferences at a fine scale (Mul et al., 2019).

**Selection Criteria:** Older female northern killer whales have been described as more difficult to approach closely for biopsy than males (Barrett-Lennard et al., 1996).

**Tagging Protocol:** Trappability in collared flycatchers, *Ficedula albicollis*, is correlated with boldness (Garamszegi et al., 2009).

**Attempts Taken to Tag and Time Taken to Tag:** Handling time, i.e., time taken to attach a telemetry device, in narwhals, *Monodon monoceros*, is a significant predictor of activity levels, energy expenditure and swimming behaviour following release (Schuert et al., 2019).

**Acclimation to Tagging:** Male wandering albatrosses, *Diomedea exulans*, react more strongly to handling than females, but recover quicker (Weimerskirch et al., 2002).

**Duration Tagged:** Roe deer, *Capreolus capreolus*, fitted with a GPS collar show significant changes in baseline behaviour for several days after capture and handling, showing a preference for refuge habitat, avoiding sources of human disturbance, and showing reduced activity levels (Morellet et al., 2009).

**Behavioural Response to Tagging:** European badgers, *Meles meles*, that respond adversely to capture are also more likely to show stronger behavioural responses to a subsequent stressor (Sun et al., 2015).

**Tagging History:** Great tits, *Parsus major*, that are caught and handled more frequently become easier to catch, have significantly higher breathing rates and are more docile in response to bleeding (Van Oers & Carere, 2007).

**Exposure**

**Sound and Exposure Characteristics:** These are clearly described in BRS and CEE studies (e.g., Bejder et al., 2009; Ellison et al., 2011, 2018; Harris et al., 2018; Houser, 2013, Southall et al., 2007, 2012, 2016, 2019).

**Proximity:** Cuvier's beaked whales, *Ziphius cavirostris*, react to simulated MFAS at short ranges but not to actual MFAS at long ranges, even at comparable levels (DeRuiter et al., 2013).

**Movement:** Avoidance behaviour and rate of humpback whales, *Megaptera novaeangliae*, is significantly affected by boat approach type (Fiori et al., 2019).

**Speed:** Indo-Pacific humpback dolphins, *Sousa chinensis*, exhibit stronger negative behavioural responses to fast-moving vessels than slower-moving vessels (Ng & Leung, 2003).

**Bearing and Orientation:** Grey whales, *Eschrichtius robustus*, will avoid a sound source (emitting low-frequency sonar signals) when it is placed directly in their migratory path but will ignore it when it is placed 2 km off their migratory path, despite similar received sound levels (Buck & Tyack, 2000; Clark et al., 1999).

**Receptor**

**Sex:** Male and female northern resident killer whales use different avoidance tactics when approached by vessels. Both respond by moving less predictably, but females tend to reduce surfacing predictability whereas males tend to reduce directional predictability (Williams et al., 2002).

**Age:** Age is a strong predictor of flight initiation distance (FID) in marine iguanas, *Amblyrhynchus cristatus* (Berger et al., 2007).

**Reproductive State:** Polar bear, *Ursus maritimus*, females with cubs react at larger distances and more strongly to approaching snowmobiles than adult males or lone adult females (Andersen & Aars, 2008).

**Nutritional State:** Ruddy turnstones, *Arenaria interpres*, in better body condition are more vigilant, take flight sooner and fly further away when approached by humans than those in poorer condition (Beale & Monaghan, 2004a).

**Behavioural State**: Deep-feeding and non-feeding blue whales, *Balaenoptera musculus*, respond strongly to similar MFAS, whereas shallow-feeding blue whales do not (Goldbogen et al., 2013).

**Social Context** **– Group Size:** In waterbirds, larger flocks are more likely to respond to a drone approach and respond at a greater distance than smaller flocks (Jarrett et al., 2020).

**Social Context – Group Composition:** Humpback whale groups with calves present are more sensitive to the presence of vessels than groups without calves (Stamation et al., 2010).

**Coping Style:** Responses to acoustic disturbance in nesting great tits is more dependent on personality and sex than noise characteristics (Naguib et al., 2013). “Bolder” parents are less affected by noise disturbance and return to their nest boxes sooner than “shyer” parents.

**Anti-Predator Strategy:** Sperm whales, *Physeter macrocephalus*, for example, respond to both 1-2 kHz sonar and killer whale vocalizations by switching to a non-foraging, non-resting state (Isojunno et al., 2016).

**Diel Patterns:** Chilean blue whales perform significantly shallower dives at night and spend more time closer to the surface than during the day, increasing their risk of ship strikes (Caruso et al., 2020).

**Seasonal Patterns:** Harbour seals, *Phoca vitulina*, during the breeding season are less alert to, less likely to flee, and quicker to return to a haul-out site (than during pre- and post-breeding seasons) when approached by humans or vessels (Andersen et al., 2012).

**Habitat Quality:** Ruddy turnstones feeding at enriched foraging sights have significantly greater FIDs than those feeding at poorer-quality sites (Beale & Monaghan, 2004a).

**Chronic Disturbance:** Magellanic penguin chicks, *Spheniscus magellanicus*, raised in tourist-visited areas of a breeding colony are less likely to flee when approached by humans than chicks raised in areas not visited by tourists (Walker et al., 2005).

**Exposure History – Focal Animal and Bystander:** Previous experience of capture in European badgers is associated with lower behavioural response scores to a subsequent stressor (Sun et al., 2015).

**Predation Pressure – Natural:** FIDs in two-banded plovers, *Charadrius falklandicus*, to human disturbance are greater at sites where predators are present (St Clair et al., 2010).

**Predation Pressure – Human:** Hunted ungulate populations show significantly greater FIDs than non-hunted populations (Stankowich, 2008).

**Applying the framework – Controlled Exposure Experiment case studies**

The framework was applied to seven cetacean species for which CEEs have been conducted: blue whales from the Southern California Behavioral Response Study (SOCAL-BRS) (Friedlaender et al., 2016; Southall et al., 2019); humpback whales from the Behavioral Response of Australian Humpback whales to Seismic Surveys (BRAHSS) (Dunlop et al., 2016, 2017); sperm whales, northern bottlenose whales, *Hyperodon ampullatus*, and long-finned pilot whales, *Globicephala melas*, from the 3S Behavioral Response Study (3S-BRS) (Miller et al., 2012, 2017; Isojunno & Miller, 2015; Isojunno et al., 2016, 2017, 2018; Wensveen et al., 2019); and Cuvier’s beaked whales, *Ziphius cavirostris*, and short-finned pilot whales, *Globicephala macrohynchus*, from the Atlantic Behavioral Response Study (Atlantic BRS) off Cape Hatteras, North Carolina.

**Supplementary References**

Andersen, M., & Aars, J. (2007). Short-term behavioural response of polar bears (Ursus maritimus) to snowmobile disturbance. *Polar Biology, 31*, 501-507.

Andersen, S. M., Teilmann, J., Dietz, R., Schmidt, N. M., & Miller, L. A. (2012). Behavioural responses of harbour seals to human-induced disturbances. *Aquatic Conservation Marine and Freshwater Ecosystems, 22*, 113-121.

Barrett-Lennard, L. G., Ford, J. K., & Heise, K. A. (1996). The mixed blessing of echolocation: differences in sonar use by fish-eating and mammal-eating killer whales. *Animal Behaviour, 51*, 553-565.

Beale, C. M., & Monaghan, P. (2004a). Behavioural responses to human disturbance: a matter of choice? *Animal Behaviour, 68,* 1065-1069.

Bejder, L., Samuels, A., Whitehead, H., & Gales, N. (2006). Interpreting short-term behavioural responses to disturbance within a longitudinal perspective. *Animal Behaviour, 72(5)*, 1149-1158.

Berger, S., Wikelski, M., Romero, L. M., Kalko, E.K., & Rödl, T. (2007). Behavioral and physiological adjustments to new predators in an endemic island species, the Galápagos marine iguana. *Hormones and Behavior, 52*, 653-663.

Buck, J. R., & Tyack, P. L. (2000). Response of gray whales to low- frequency sounds. *Journal of the Acoustical Society of America, 107,* 2774.

Caruso, F., Hickmott, L., Warren, J. D., Segre, O., Chiang, G., Bahamonde, P., Espanol-Jimenez, S., Li, S., & Bocconcelli, A. (2020). Diel differences in blue whale (*Balaenoptera musculus*) dive behaviour increase nighttime risk of ship strikes in northern Chilean Patagonia. *Integrative Zoology, 0*, 1-18.

Clark, C. W., Tyack, P., & Ellison, W. T. (1999). *Responses of four species of whales to sounds of SURTASS LFA sonar transmissions*. Cornell University, Ithaca, New York; Woods Hole Oceanographic Institution, Woods Hole, Massachusetts, and Marine Acoustics, Arlington, Virginia.

DeRuiter, S. L., Southall, B. L., Calambokidis, J., Zimmer, W. M., Sadykova, D., Falcone, E. A., Friedlaender, A. S., Joseph, J. E., Moretti, D., Schorr, G. S., & Thomas, L., (2013). First direct measurements of behavioural responses by Cuvier's beaked whales to mid-frequency active sonar. *Biology Letters, 9(4)*, 20130223.

Dunlop, R. A., Noad, M. J., McCauley, R. D., Kniest, E., Slade, R., Paton, D., & Cato, D. H. (2016). Response of humpback whales (*Megaptera novaeangliae*) to ramp-up of a small experimental air gun array. *Marine Pollution Bulletin, 103(1-2)*, 72-83.

Dunlop, R. A., Noad, M. J., McCauley, R. D., Scott-Hayward, L., Kniest, E., Slade, R., Paton, D., & Cato, D.H. (2017). Determining the behavioural dose–response relationship of marine mammals to air gun noise and source proximity. *Journal of Experimental Biology,* *220,* 2878-2886.

Ellison, W., Southall, B., Clark, C., & Frankel, A. (2011). A new context-based approach to assess marine mammal behavioral responses to anthropogenic sounds. *Conservation Biology, 26*, 21-28.

Fiori, L., Martinez, E., Orams, M. B., & Bollard, B. (2019). Effects of whale-based tourism in Vava’u, Kingdom of Tonga: Behavioural responses of humpback whales to vessel and swimming tourism activities. *PLoS ONE, 14(7),* e0219364.

Friedlaender, A. S., Hazen, E., Goldbogen, J., Stimpert, A., Calambokidis, J., & Southall, B. (2016). Prey-mediated behavioural responses of feeding blue whales in controlled sound exposure experiments. *Ecological Applications, 26(4),* 1075-1085.

Garamszegi, L. Z., Eens, M., & Török, J. (2009). Behavioural syndromes and trappability in free-living collared flycatchers, *Ficedula albicollis*. *Animal Behaviour, 77*, 803-812.

Goldbogen, J. A., Southall, B. L., DeRuiter, S. L., Calambokidis, J., Friedlaender, A. S., Hazen, E. L., Falcone, E. A., Schorr, G. S., Douglas, A., & Moretti, D.J. (2013). Blue whales respond to simulated mid-frequency military sonar. *Proceedings of the Royal Society of London B: Biological Sciences, 280*, 20130657.

Harris, C. M., Thomas, L., Falcone, E. A., Hildebrand, J., Houser, D., Kvadsheim, P.H., Lam, F. P. A., Miller, P. J., Moretti, D. J., & Read, A.J. (2018). Marine mammals and sonar: Dose-response studies, the risk-disturbance hypothesis and the role of exposure context. *Journal of Applied Ecology, 55*, 396-404.

Houser, D.S., Martin, S.W., & Finneran, J.J. (2013). Exposure amplitude and repetition affect bottlenose dolphin behavioral responses to simulated mid-frequency sonar signals. *Journal of Experimental Marine Biology and Ecology*, *443*, 123–133.

Isojunno, S., & Miller, P.J. (2015). Sperm whale response to tag boat presence: biologically informed hidden state models quantify lost feeding opportunities. *Ecosphere,* *6,* 1-46.

Isojunno, S., Aoki, K., Curé, C., Kvadsheim, P.H., & Miller, P. J. O. M. (2018). Breathing patterns indicate cost of exercise during diving and response to experimental sound exposures in long-finned pilot whales. *Frontiers in Physiology,* *9,* 1462.

Isojunno, S., Curé, C., Kvadsheim, P. H., Lam, F. P. A., Tyack, P. L., Wensveen, P. J., & Miller, P. J. O. M. (2016). Sperm whales reduce foraging effort during exposure to 1–2 kHz sonar and killer whale sounds. *Ecological Applications, 26*, 77-93.

Isojunno, S., Sadykova, D., DeRuiter, S., Curé, C., Visser, F., Thomas, L., Miller, P., & Harris, C. (2017). Individual, ecological, and anthropogenic influences on activity budgets of long‐finned pilot whales. *Ecosphere,* *8,* e02044.

Jarrett, D., Calladine, J., Cotton, A., Wilson, M. W. & Humphreys, E. (2020). Behavioural responses of non-breeding waterbirds to drone approach are associated with flock size and habitat. *Bird Study, 67*, 190-196.

Miller, P., Siegal, E., Narazaki, T., Wensveen, P.J., Isojunno, S., Woo, K., Hooker, S.K., van IJsselmuide, S., & Hansen, R. (2017). The northern bottlenose whale (*Hyperoodon ampullatus*) of Jan Mayen. *The Society for Marine Mammalogy’s 22nd Biennial Conference on the Biology of Marine Mammals, Abstract Book*. Halifax, Nova Scotia, Canada.

Miller, P. J. O., Kvadsheim, P. H., Lam, F. P. A., Wensveen, P. J., Antunes, R., Alves, A. C., Visser, F., Kleivane, L., Tyack, P. L., & Sivle, L. D. (2012). The Severity of Behavioral Changes Observed During Experimental Exposures of Killer (*Orcinus orca*), Long-Finned Pilot (*Globicephala melas*), and Sperm (*Physeter macrocephalus*) Whales to Naval Sonar. *Aquatic Mammals,* *38,* 362-401.

Morellet, N., Verheyden, H., Angibault, J. M., Cargnelutti, B., Lourtet, B., & Hewison, M. A. (2009). The effect of capture on ranging behaviour and activity of the European roe deer *Capreolus capreolus*. *Wildlife Biology, 15*, 278-287.

Mul, E., Blanchet, M., Biuw, M., & Rikardsen, A. (2019). Implications of tag positioning and performance on the analysis of cetacean movement. *Animal Biotelemetry, 7,* 11.

Naguib, M., van Oers, K., Braakhuis, A., Griffioen, M., de Goede, P., & Waas, J. R. (2013). Noise annoys: effects of noise on breeding great tits depend on personality but not on noise characteristics. *Animal Behaviour, 85*, 949-956.

Ng, S. L., & Leung, S. (2003). Behavioural response of Indo-Pacific humpback dolphin (*Sousa chinensis*) to vessel traffic. *Marine Environmental Research, 56,* 555-567.

Schuert, C. R., Marcoux, M., Hussey, N. E., Watt, C. A., & Auger-Méthé, M. (2021). Assessing the post-release effects of capture, handling and placement of satellite telemetry devices on narwhal (*Monodon monoceros*) movement behaviour. *Conservation Physiology, 9(1)*, coaa128.

Southall, B. L., Bowles, A. E., Ellison, W. T., Finneran, J. J., Gentry, R. L., Greene, C. R. J., Kastak, D., Ketten, D. R., Miller, J. H., Nachtigall, P. E., Richardson, W. J., Thomas, J. A., & Tyack, P. L. (2007). Marine mammal noise exposure criteria: initial scientific recommendations. *Aquatic Mammals, 33*, 411-414.

Southall, B. L., Moretti, D., Abraham, B., Calambokidis, J., DeRuiter, S. L., & Tyack, P. L. (2012). Marine Mammal Behavioral Response Studies in Southern California: Advances in Technology and Experimental Methods. *Marine Technology Society Journal, 46*, 48-59.

Southall, B. L., Nowacek, D. P., Miller, P. J. O., & Tyack, P. L. (2016). Experimental field studies to measure behavioural responses of cetaceans to sonar. *Endangered Species Research, 31*, 293-315.

Southall, B., Quick, N., Hastie, G., Tyack, P., & Boyd, I. (2017). Mitigation of harm during a novel behavioural response study involving active sonar and wild cetaceans. *Journal of Cetacean Research and Management, 16,* 29-38.

Southall, B. L., DeRuiter, S. L., Friedlaender, A., Stimpert, A. K., Goldbogen, J. A., Hazen, E., Casey, C., Fregosi, S., Cade, D. E., Allen, A. N., Harris, C. M., Schorr, G., Moretti, D., Guan, S., & Calambokidis, J. (2019). Behavioural responses of individual blue whales (Balaenoptera musculus) to mid-frequency military sonar. *Journal of Experimental Biology, 222(5)*, jeb190637.

Clair, J. J., García-Peña, G. E., Woods, R. W., & Székely, T. (2010). Presence of mammalian predators decreases tolerance to human disturbance in a breeding shorebird. *Behavioral Ecology, 21*, 1285-1292.

Stamation, K. A., Croft, D. B., Shaughnessy, P. D., Waples, K. A., & Briggs, S. V. (2010). Behavioral responses of humpback whales (Megaptera novaeangliae) to whale-watching vessels on the southeastern coast of Australia. *Marine Mammal Science, 26*, 98-122.

Stankowich, T. (2008). Ungulate flight responses to human disturbance: a review and meta-analysis. *Biological Conservation, 141*, 2159-2173.

Sun, Q., Stevens, C., Newman, C., Buesching, C., & Macdonald, D. (2015). Cumulative experience, age-class, sex and season affect the behavioural responses of European badgers (*Meles meles*) to handling and sedation. *Animal Welfare, 24*, 373-385.

Van Oers, K., & Carere, C. (2007). Long-term effects of repeated handling and bleeding in wild caught great tits Parus major. *Journal of Ornithology, 148*, 185-190.

Walker, B. G., Boersma, P. D., & Wingfield, J. C. (2005). Physiological and behavioral differences in Magellanic penguin chicks in undisturbed and tourist-visited locations of a colony. *Conservation Biology, 19*, 1571-1577.

Weimerskirch, H., Shaffer, S. A., Mabille, G., Martin, J., Boutard, O., & Rouanet, J. L. (2002). Heart rate and energy expenditure of incubating wandering albatrosses: basal levels, natural variation, and the effects of human disturbance. *Journal of Experimental Biology, 205*, 475-483.

Wensveen, P. J., Isojunno, S., Hansen, R. R., von Benda-Beckmann, A. M., Kleivane, L., van IJsselmuide, S., Lam, F. P. A., Kvadsheim, P. H., DeRuiter, S. L., & Curé, C. (2019). Northern bottlenose whales in a pristine environment respond strongly to close and distant navy sonar signals. *Proceedings of the Royal Society B, 286,* 20182592.

Williams, R., Trites, A. W., & Bain, D. E. (2002). Behavioural responses of killer whales (*Orcinus orca*) to whale-watching boats: opportunistic observations and experimental approaches. *Journal of Zoology, 256*, 255-270.
